# HIV-1C in-House RNA-Based Genotyping Assay for Detection of Drug Resistance Mutations in Samples with Low-Level Viral Loads

**DOI:** 10.2147/IDR.S388816

**Published:** 2022-12-22

**Authors:** Ontlametse T Bareng, Wonderful T Choga, Segomotso T Maphorisa, Sekgabo Seselamarumo, Kaelo K Seatla, Patrick T Mokgethi, Dorcas Maruapula, Mompati L Mogwele, Doreen Ditshwanelo, Natasha O Moraka, Irene Gobe, Modisa S Motswaledi, Joseph M Makhema, Rosemary Musonda, Roger Shapiro, Max Essex, Vlad Novitsky, Sikhulile Moyo, Simani Gaseitsiwe

**Affiliations:** 1 Botswana Harvard AIDS Institute Partnership, Gaborone, Botswana; 2 School of Allied Health Professions, Faculty of Health Sciences, University of Botswana, Gaborone, Botswana; 3 Ministry of Health and Wellness, Republic of Botswana, Gaborone, Botswana; 4 Department of Biological Sciences, Faculty of Science, University of Botswana, Gaborone, Botswana; 5 Department of Biological Science and Biotechnology, Botswana International University of Science and Technology, Palapye, Botswana; 6 Department of Immunology and Infectious Diseases, Harvard T.H. Chan School of Public Health, Boston, MA, USA

**Keywords:** in-house genotyping, low-level viremia, samples, HIV-1C drug resistance testing

## Abstract

**Purpose:**

Monitoring HIV-1 drug resistance mutations (DRM) in treated patients on combination antiretroviral therapy (cART) with a detectable HIV-1 viral load (VL) is important for the selection of appropriate cART. Currently, there is limited data on HIV DRM at low-level viremia (LLV) (VL 401–999 copies/mL) due to the use of a threshold of VL ≥1000 copies/mL for HIV DRM testing. We here assess the performance of an in-house HIV drug resistance genotyping assay using plasma for the detection of DRM at LLV.

**Methods:**

We used a total of 96 HIV plasma samples from the population-based Botswana Combination Prevention Project (BCPP). The samples were stratified by VL groups: 50 samples had LLV, defined as 401–999 copies/mL, and 46 had ≥1000 copies/mL. HIV pol (PR and RT) region was amplified and sequenced using an in-house genotyping assay with BigDye sequencing chemistry. Known HIV DRMs were identified using the Stanford HIV Drug Resistance Database. Genotyping success rate between the two groups was estimated and compared using the comparison of proportions test.

**Results:**

The overall genotyping success rate was 79% (76/96). For VL groups, the genotyping success was 72% (36/50) at LLV and 87% (40/46) at VL ≥1000 copies/mL. Among generated sequences, the overall prevalence of individuals with at least 1 major or intermediate-associated DRM was 24% (18/76). The proportions of NNRTI-, NRTI- and PI-associated resistance mutations were 28%, 24%, and 0%, respectively. The most predominant mutations detected were K103N (18%) and M184V (12%) in NNRTI- and NRTI-associated mutations, respectively. The prevalence of DRM was 17% (6/36) at LLV and 30% (12/40) at VL ≥1000 copies/mL.

**Conclusion:**

The in-house HIV genotyping assay successfully genotyped 72% of LLV samples and was able to detect 17% of DRM amongst them. Our results highlight the possibility and clinical significance of genotyping HIV among individuals with LLV.

## Introduction

A subset of people with human immunodeficiency virus (PWH) on potent antiretroviral therapy (ART) have low-level viremia (LLV), a detectable HIV viral load (VL) below 1000 copies/mL.^1^,^2^ Lack of HIV suppression may be attributed to many factors such as the presence of drug resistance mutations (DRM), metabolic complications affecting the pharmacokinetics of the drugs, and lack of medication adherence.^2–4^ Recent studies have found the presence of DRMs in LLV to be a strong predictor of subsequent virologic failure (VF).^5–8^ Despite these recent findings, no clear conclusion can be drawn on the impact of DRM on individuals with LLV and its long-term clinical outcomes because of the limited availability of HIV drug resistance genotyping assays validated for samples with LLV.

Most commercial RNA-based genotyping assays require HIV plasma viral loads > 1000 copies/mL for increased amplification success rates and accurate results.^9^,^10^ The other reason for limited data on DRM at LLV is linked to the threshold of 1000 copies/mL to define virological failure and threshold for genotyping testing, especially in low and middle-income settings. HIV proviral DNA is very useful in LLV or when plasma sequencing is not successful.^11–13^ However, proviral DNA-based assays are not routinely utilized for drug resistance testing for clinical monitoring purposes, which highlights the need for optimization of RNA-based genotyping assays for LLV. There is the possibility that genotyping samples with low VL using RNA-based approaches are limited by small copy numbers, which may render inaccurate genotyping results. However, there are optimized HIV genotyping assays for LLV with high success rates which utilize RNA-based approaches.^10^,^14–17^ The ability to genotype LLV could contribute to improved management of PWH experiencing low-level viremia, especially for the selection of appropriate ART, preserving future ART options. Therefore, our study aimed to optimize and assess the performance of an in-house RNA-based HIV genotyping assay for LLV in HIV-1 subtype C which predominates in Botswana and is the most prevalent HIV-1 subtype globally. Botswana is one of the countries with a high HIV prevalence of 18.6% among 15–49-year-olds with 7200 individuals newly infected in 2021.^18^ However, Botswana has had major successes in the country-wide implementation of antiretroviral therapy (ART) and high viral load suppression rates.^19^ The country has reached UNAIDS 95–95-95 UNAIDS targets where 95.1% of the population is aware of their HIV status, 98.0% are on ART and 97.9% are virally suppressed.^20^ Despite major advances in the development of antiretroviral (ARV) drugs and ARV therapy (ART) treatment guidelines,^21–23^ Botswana like other middle-income countries continues to face challenges including development, transmission and spread of HIV drug resistance mutations.^24^ This highlights the need for HIV drug resistance monitoring at any detectable VL with LLV included.

## Methods

### Study Population

Stored plasma samples from whole blood collected from PWH aged 16–64 years who were enrolled in the Botswana Combination Prevention Project (BCPP) from 2013 to 2018 and residing in 30 communities across central, northern, and southern parts of Botswana were utilized. BCPP was a community-randomized trial evaluating whether a package of standard HIV prevention trials would lessen the frequency of HIV cases over time among ART-experienced and -naïve individuals in Botswana, as previously described.^19^,^25^

### Selection of Study Samples

Abbott m2000sp/rt (Abbott Laboratories, Abbott Park, IL, USA) was used for the quantification of HIV-1 VL with the lowest limit of detection at 40 copies/mL and the highest limit at 10,000,000 copies/mL. A total of 96 samples with VL > 400 copies/mL and enough volume ≥ 200μL were conveniently sampled from remnants of the BCPP cohort for the assay optimization, with 52% at LLV and 48% at VL ≥ 1000 copies/mL. Samples with VL 401–999 copies/mL were categorized as LLV, while those with VL ≥ 1000 copies/mL were considered to be in virologic failure (VF),^26^ per WHO guidelines.

### Nucleic Acid Purification

HIV RNA was manually extracted from 200μL plasma samples stored at −80°C using the Zymo Research Quick-RNA Viral kit (Zymo Research, Pretoria, South Africa) as per the manufacturer’s instructions. The isolated RNA was used as a first-round PCR template and stored at −80°C for future use.

### Amplification of HIV Pol

The HIV Pol (PR and RT) region of 1.1 kb, spanning the entire protease and the first 800 bps of RT, was amplified with a transcriptor one-step RT-PCR kit (Roche Applied Science, Penzberg, Germany). A one-step RT-PCR reaction mixture was prepared to consist of 0.5µL Transcriptase Enzyme Roche One Step, 7µL of RNase-free water, 5µL of transcriptor one-step RT-PCR 5X Buffer, 2.5µL mixture of primers CWF1-LNA2(5’-GAA GGA CCA AAT GAA AGA YTG-3’ (2 μM) and CWR1-LNA3 (5’-GCA TAC TTY CCG TTT TCA G-3’ (2 μM), and 10µL of HIV RNA, making a total of 25µL. The PCR parameters for the initial one-step PCR were reverse transcribed at 50°C for 30 minutes, initial denaturation at 94°C for 7 mins, 10 cycles consisting of a denaturation stage at 94°C for 10 seconds, annealing stage of 55°C for 30 seconds and an extension step of 68°C for 2 minutes and then 35 cycles with denaturation at 94°C for 10 seconds, annealing stage of 55.5°C for 30 seconds and final extension step of 68°C for 2 minutes, increasing each cycle by 10 seconds. The last stage was the final elongation at 68°C for 5 minutes with a hold stage at 4°C for a maximum of 18 hours. The nested PCR was performed using 1µL aliquot of first-round PCR product mixed with 9.0µL of RNase-free water, 12.5µL of Phusion High-Fidelity PCR Master Mix with HF Buffer, and 2.5µL of primers CWF1-LNA2(5’-GAA GGA CCA AAT GAA AGA YTG-3’ (2 μM) and RT-20C (5’CTG CCA ATT TCT AAC TGC CTT C-3 ‘(2 μM), making 25µL of reaction volume. The PCR parameters for the nested PCR were initial denaturation at 98°C for 30 seconds, 35 cycles made of denaturation at 98°C for 10 seconds, an annealing stage of 62°C for 30 seconds, an extension step of 72°C for 20 seconds, and the final elongation at 72°C for 10 minutes with a hold stage at 4°C for a maximum of 18 hours. Amplification was confirmed by electrophoresis in a 1% agarose gel in Tris-Borate-EDTA (TBE) buffer and ran at 90 volts for 45 minutes stained with 5μL ethidium bromide (0.5 mg/mL) and visualized under a UV source (260 nm). Failed samples were re-extracted and PCR amplification was repeated once using a set of rescue primers (Table 1) with the same PCR parameters and conditions.

**Table 1:** Rescue Primers Used When Samples Failed to Amplify

Primer Name	Sequence (5’-3’)	Purpose
PRTM-F1**	F1a-TGAARGAITGYACTGARAGRCAGGCTAAT F1b-ACTGARAGRCAGGCTAATTTTTTAG	Mixture of 2 primers for RT-PCR
RT-R1	ATCCCTGCATAAATCTGACTTGC	RT-PCR
PRT-F2	CTTTARCTTCCCTCARATCACTCT	Nested PCR
RT-R2	CTTCTGTATGTCATTGACAGTCC	Nested PCR
SeqF3	AGTCCTATTGARACTGTRCCAG	Sequencing
SeqR3	TTTYTCTTCTGTCAATGGCCA	Sequencing
SeqF4	CAGTACTGGATGTGGGRGAYG	Sequencing
SeqR4	TACTAGGTATGGTAAATGCAGT	Sequencing

**Note**: **A mixture of primers F1a and F1b.

### Sequencing

Successfully amplified HIV pol was purified using Exo-CIP™ Rapid PCR Clean-up Kit (New England Biolabs, Ipswich, USA) according to the manufacturer’s instructions. The purified amplicons were sequenced using BigDye Terminator v3.1 Cycle Sequencing Kit (Applied Biosystems, Foster City, USA) on a 3031xl genetic analyzer using primers CWF1-LNA2(5’-GAA GGA CCA AAT GAA AGA YTG-3’), CWCS2(5’-AGAACTCAAGACTTTTGGG-3’), CWCS3(5’-TGCTGGGTGCGGTATTC-3’), CWCS5(5’TGGTAAATTTGATGTCCAT-3’), Seq2.1(5’GGCCAGGAATTTTCTTCAGAGC-3’), Seq6(5’-CCATCCCTGTGGAAGCACATTA-3’) and RT-20C (5’CTG CCA ATT TCT AAC TGC CTT C-3’). The cycle sequencing reaction mix contained 4.8μL of RNase-free water, 3μL of Big Dye 5X sequencing buffer, 1μL BigDye terminator, 0.2 μL of 2 μM of each sequencing primer, and 1μL of the purified amplicon, to make a total reaction volume of 10μL. The cycle sequencing reaction conditions were as follows: 25 cycles at 96°C for 10 seconds, 50°C for 5 seconds, 60°C for 4 minutes and hold at 4°C. The BigDye XTerminator purification kit (Applied Biosystems, Foster City, USA) was used to purify the sequencing reaction by adding 10μL of the BigDye XTerminator and 45μL SAM solution to cycle sequencing products. The reaction plate was vortexed at 1800 rpm for 45 minutes and then centrifuged at 3000g for 3 minutes at room temperature. Next, 65μL of purified cycle sequencing products in a reaction plate were analyzed using an ABI 3031xl genetic analyzer (Applied Biosystems, Foster City, USA)

### Bioinformatics Analysis

The quality and read length of sequences obtained from the ABI 3130XL Genetic Analyzer were assessed using Sequencer Version 5.0 by manually trimming the beginning and end of each sequence to remove ambiguous nucleotides. Only sequences with quality scores of ≥73% were assembled into a contiguous sequence (contig). Mixed bases or ambiguous nucleotides were adopted or confirmed with the sequences covering the same position in the contig. The same software was used to assemble multiple reads of each sequence into a single contig(consensus sequence). The targeted sequence length was 1.1 kb, covering the partial Pol region. Sequence imputation and quality control assessment was assessed using AliView v1.26. Reference-based multiple sequence alignment (MSA) was constructed using muscle v3.8.31 implemented in AliView v1.26 and HIV-1 reference strain sequence (HXB2). MSA was further used for downstream analyses including phylogenetic inferences based on maximum-likelihood (ML), clade assessment, and DRM analysis.

All generated sequences that passed the QC were included in the phylogenetic tree. A maximum-likelihood tree topology was inferred from the reference-based MSA in IQ-TREE2 using General Time Reverse plus F plus R4 (GTR+F+R4) as a best-fit model of nucleotide substitution. A total of 1000 bootstrap replicates were employed to infer support for branches in the resulting tree topology using Booster. Tree visuals and annotations were performed in FigTree v1.4.4. Posterior probabilities 0.90 and above were noted as statistically significant.

### HIV Drug Resistance Analysis

The MSA was imported into the Stanford HIV Drug Resistance Database to determine the HIV drug resistance profiles of each of the consensus sequences. HIV pol region was analysed for known DRM associated with nucleoside reverse transcriptase inhibitors (NRTIs), non-nucleoside reverse transcriptase inhibitors (NNRTIs) and protease inhibitors (PIs) according to the Stanford University HIV Drug Resistance Database.^27^ In sequences generated from ART naïve individuals, Calibrated Population Resistance in the Stanford University HIV database (https://hivdb.stanford.edu/cpr/form/PRRT/) was used to identify known surveillance drug resistance mutations.

### Statistical Analysis

Patients’ general characteristics among the two VL groups were compared using Wilcoxon rank-sum test for continuous variables and Fisher’s exact test for categorical variables. The success rate of genotyping was calculated using the number of successfully genotyped samples in each VL category over the total number in that category multiplied by 100%. Genotyping success rates at LLV and VL ≥1000 copies/mL were compared using a comparison of proportions test to determine any statistical difference in the performance of the assay between these two groups. The prevalence of mutations was estimated with 95% confidence intervals using the binomial exact method for each group and further compared between the two VL groups. All of the analyses were done using STATA version 15 and p-values < 0.05 were considered statistically significant.

## Results

### Baseline and Clinical Characteristics

Among 96 participants included, 72% were females and the median age for all participants was 33.5, IQR: of 26.4–41 years. About 67% were antiretroviral therapy-experienced and the median HIV VL was 982, IQR: 669–12,422 copies/mL. The study participants were stratified into two VL groups: LLV (VL: 401–999 copies/mL) and VL ≥ 1000 copies/mL. Between these 2 groups, there was a statistically significant difference in ART status, whereby 64% of the LLV group were ART naïve and all participants with VL ≥ 1000 copies/mL were on ART (Table 2).

**Table 2:** Baseline Demographics and Clinical Characteristics for Study Participants

Variable	Total n=96 (%)	LLV (401–999 Copies/mL) n=50 (%)	VL>1000 Copies/mL n=46 (%)	*P-values*
**Gender, n (%)**				
**Female**	69 (72)	38 (76)	31 (67)	*0.37*^a^
**Male**	27 (28)	12 (24)	15 (37)	
**Median Age in years** **(Q1, Q3)**	33.5 (26.4, 41)	35.7 (27, 40.8)	31.8 (25.1, 42.7)	*0.43*
**ART Status**				
**Experienced**	64 (67)	18 (36)	46 (100)	*<0.01*^a^
**Naïve**	32 (33)	32 (64)	0	
**Median Viral Load in copies/mL (Q1, Q3)**	982 (669–12,422)	699 (528–872)	14,004 (2561–47,521)	*<0.01*

**Notes**: ^a^P-values were obtained by Fishers’ test while other p-values were from Wilcoxon rank sum test.

**Abbreviations**: ART, antiretroviral therapy; LLV, low level viremia; n, number; VL, viral load; Q1, first quartile; Q3, third quartile.

### Amplification and Sequencing Success Rate

**Figure 1:**
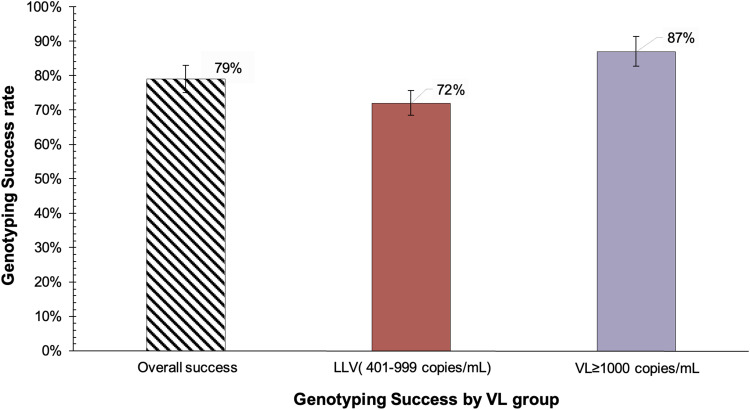
Genotyping success of in-house RNA-based HIV genotyping assay, overall and stratified by VL group.

**Abbreviations**: LLV, low-level viremia (VL 401–999 copies/mL); VL, viral load.

**Figure 2:**
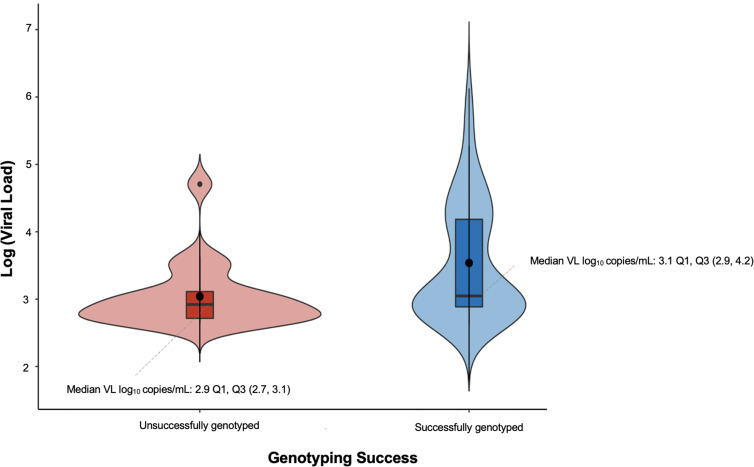
Association of genotyping outcomes with viral load measurement.

**Abbreviations**: Q1, first quartile; Q3, third quartile; VL, viral load.

The lowest VL that the assay was able to successfully genotype was 423 copies/mL. The overall genotyping success rate was 79% (Figure 1), 69% (66/96) of samples were successfully genotyped by the original protocol while 10% (10/96) were genotyped using the rescue protocol described in Table 1. Stratifying by the VL group, a total of 72% (36/50) of samples with VL 401–999 copies/mL were successfully genotyped, compared to 87% (40/46) at VL ≥ 1000 copies/mL (p=0.07). The median log VL of successfully genotyped samples was 3.1 VL log_10_ copies/mL, IQR: 2.9–4.2, while the median for the failed samples was 2.9 VL log_10_ copies/mL, IQR: 2.7–3.1 (Figure 2). The genotyping outcomes of the in-house genotyping assay were not associated with the viral load (p=0.2).

### Phylogenetic Analysis

**Figure 3:**
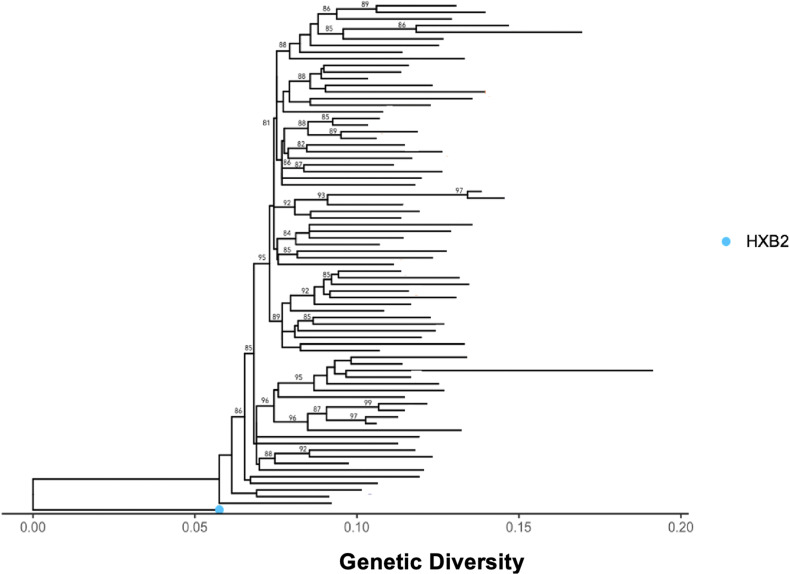
Maximum likelihood phylogenetic tree constructed using 1000 bootstrap values showing the uniqueness of the sequences and that there is no clustering of sequences by their VL group. The light-blue dot represents standard HIV reference strain.

**Abbreviation**: HXB2, standard HIV reference strain.

Sequence quality control and clustering were performed using phylogenetic analysis. Using pairwise distance analysis, the 76 sequences generated by the assay were unique. The maximum likelihood phylogenetic tree was constructed from HIV-1C RT/PR sequences generated using our in-house genotyping assay (Figure 3). There was no clustering of sequences by their VL group.

### Drug Resistance Patterns

**Figure 4:**
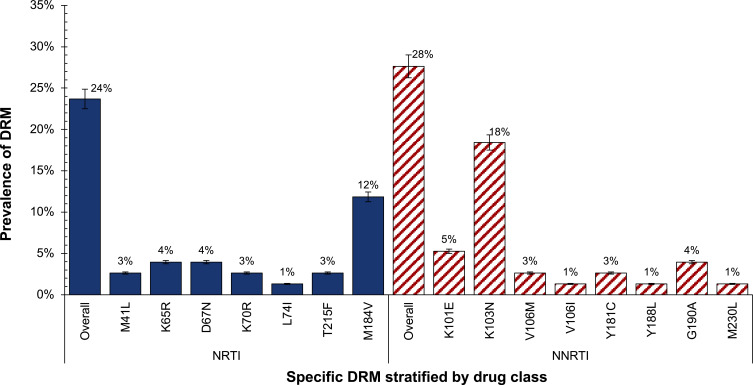
HIV DRM classified by NRTI and NNRTI resistance classes (N=76) using an in-house RNA-based HIV genotyping assay with detectable plasma HIV-1 RNA > 400 copies/mL.

**Abbreviations**: DRM, drug resistance mutations; NRTI, nucleotide reverse transcriptase inhibitors; NNRTI, non-nucleotide reverse transcriptase inhibitors.

**Figure 5:**
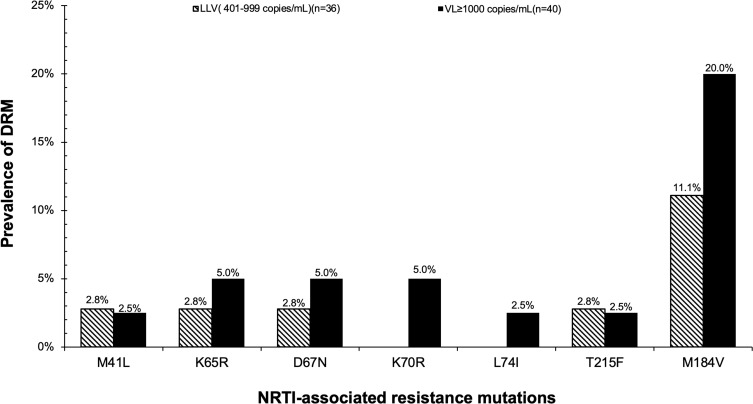
NRTI-associated resistance mutations stratified by VL groups.

**Abbreviations**: DRM, drug resistance mutations; LLV, low-level viremia; NRTI, nucleoside reverse transcriptase inhibitors; VL, viral load.

**Figure 6:**
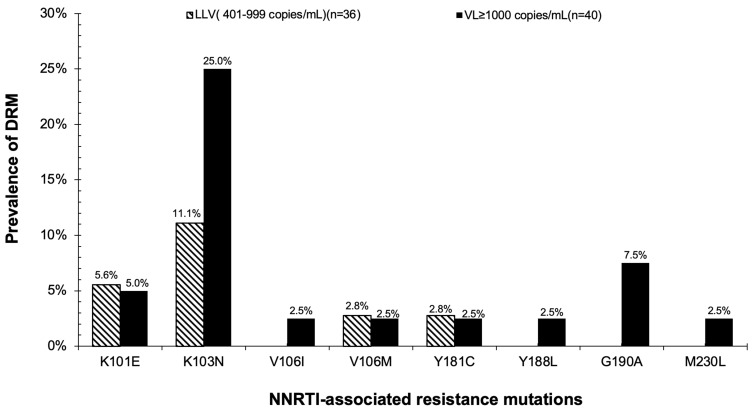
NNRTI-associated resistance mutations are stratified by VL groups.

**Abbreviations**: DRM, Drug resistance mutations; LLV, low level viremia; NNRTI, non-nucleoside reverse transcriptase inhibitors; VL, viral load.

Among the generated sequences, the overall prevalence of individuals with at least 1 major-associated DRM was 24% (18/76) (Table 3). By VL group, individuals with LLV who were both ART naïve and experienced had a 17% prevalence of DRM compared to 30% among those with VL ≥ 1000 copies/mL who were all on ART (p-value=0.18). In the LLV group, 69% (25/36) of successfully genotyped individuals were ART naïve of whom 2 had DRM (1 with only NNRTI -associated mutations K101E and K103N while another one had only NRTI-associated mutation M41L). The prevalence of DRMs were NNRTI (28%), NRTI (24%) and PI (0%)-associated resistance mutations. Only 22% (4/18) had NRTI-, 39% (7/18) had NNRTI-, and 78% (14/18) had both NNRTI- and NRTI-associated resistance mutations. The most predominant mutations detected were K103N (18%) and M184V (12%) in NNRTI- and NRTI-associated mutations, respectively (Figure 4). Both NRTI- and NNRTI-associated resistance mutations were stratified by the VL group (Figures 5 and 6).

**Table 3:** Prevalence of HIV DRM, Overall and by VL Groups

Resistance Measure	Total n=76	VL ≥ 1000 Copies/mL n=40	LLV (401–999 Copies/mL) n=36	p-value
**Any drug resistance mutation, n^a^ (%)**	18 (24%)	12 (30%)	6 (17%)	0.18
	95% CI:15–35	95% CI:17–46	95% CI: 6–33	
**Resistance category, n^b^ (%)**				
**NNRTI**	21 (28%)	15 (38%)	6 (17%)	0.04
**NRTI**	18 (24%)	12 (30%)	6 (17%)	0.18

**Notes**: ^a^Number of participants with at least 1 mutation associated with either of 2 drug classes (NNRTI or NRTI). ^b^Number of participants with at least 1 mutation in any specific drug resistance class. A comparison of proportion test was used to test the difference in the prevalence of DRM between 2 VL groups. Proportions with 95% Confidence Intervals (CI) were estimated using the Binomial exact method. Note that the sum of the number of persons with individual classes of mutations may exceed the number of persons with any mutation, as some people had more than 1 class of DRM. Note that the prevalence of PI-associated resistance mutations was not predicted.

**Abbreviations**: CI, 95% confidence intervals; LLV, low-level viremia (VL 401–999 copies/mL) in both ART-naïve and ART-experienced individuals; VL, viral load; NRTI, nucleoside reverse transcriptase inhibitor-associated mutations; NNRTI, non-nucleoside reverse transcriptase inhibitor-associated mutations.

## Discussion

Our in-house RNA-based HIV genotyping assay showed that routine HIV genotyping of low-level viremia can be performed with a success rate of 72% and with a lower limit of genotyping of 423 copies/mL. The assay was able to detect 17% of LLV participants with at least one drug resistance mutation.

The performance of the in-house genotyping assay was higher (87%) among samples with VL ≥ 1000 copies/mL, compared to the LLV group (72%), however this difference was not statistically significant (p=0.07). Our study findings highlight the potential of the assay to genotype samples with LLV and a similar trend was reported in other studies.^10^,^16^,^28–30^ It is difficult to compare our study with others on how a lower LLV range determines genotyping success due to the use of LLV 401–999 copies/mL when other studies went as low as 51 copies/mL. The same threshold of VL > 400 copies/mL was utilized in a South African cohort where the overall genotyping success rate was 83%,^31^ which was not statistically different from the 79% reported in our study (p=0.13).

Although our study had a modest sample size, the genotyping success was similar to 72.4% among 1072 samples^28^ and 67% among 756 samples, in two studies done in France.^32^ However, our genotyping success rate was relatively lower compared to other assays utilized in LLV settings with success ≥ 95%.^10^,^15^,^16^,^30^,^33^,^34^ A higher plasma input volume of ≥ 1mL is usually recommended for LLV samples, to increase HIV copies for amplification;^35–38^ however, our assay was able to successfully genotype about 72% of LLV samples using a plasma input volume of 200 µL for extraction. Nevertheless, this is the first LLV genotyping assay to have a success rate of 72% with non-concentrated virus copies using lower sample input volume.

Current HIV treatment guidelines on genotyping of detectable VL below 1000 copies/mL testing are unclear because it might be unsuccessful.^39^ Furthermore, these guidelines recommend strongly against testing for patients with VL 200–1000 copies/mL using RNA-based approaches, but rather recommend using proviral DNA. Additionally, resistance assay kits are only validated to test samples with VL ≥ 1000^40^,^41^ or VL ≥ 2000^42^,^43^ copies/mL. However, our results indicate that resistance testing of samples with viral loads below 1000 copies/mL provides clinically relevant information.

The association between VL and the overall prevalence of DRM was statistically similar when compared among individuals with LLV who were both ART naïve and experienced (17%) against those with VL ≥ 1000 copies/mL (30%) who were all on ART. However, a statistically higher prevalence of NNRTI-associated resistance was reported among individuals with VL ≥ 1000 copies/mL compared to LLV. The overall prevalence of DRM among individuals with LLV included 69% (25/36) of successfully genotyped individuals who were ART naïve, which might have lowered the NNRTI-associated resistance in this group. The main objective was to assess the practicability of genotyping LLV samples. Therefore, both ART-naïve and -experienced individuals were included in the study. We report no statistically significant difference in the prevalence of NNRTI-associated resistance mutations between 2 VL groups when ART-experienced LLV was compared to VL ≥ 1000 copies/mL. These findings were concordant with our previous study that reported no association between VL and DRM in the same cohort, although the DRMs were detected in proviral DNA.^44^ Most studies have reported concordance in DRM detected in both viral RNA and proviral DNA;^45^,^46^ therefore both can be useful in LLV for the detection of DRM.

The assay was not assessed on non-HIV-1C viruses because the HIV-1C epidemic is predominant in sub-Saharan Africa, which may make the assay applicable in the region. One of the study limitations is that the performance of the optimized assay was not compared to any commercial HIV genotyping assay, however, most of the commercial genotyping assays are validated for VL measurements of ≥1000 copies/mL which makes the comparison difficult in LLV samples. One of the study limitations is that most of the ART-experienced individuals have missing ART regimen information. In BCPP cohort, 60% of PWH were on efavirenz (EFV), 26.6% on nevirapine (NVP) and 7% on dolutegravir (DTG)-based regimens while 5% were on second-line therapy and 1% on salvage therapy among individuals who had available ARV regimen information. Those who were on EFV and NVP, were also on 2 NRTI regimens such as lamivudine (3TC) and zidovudine (ZDV) or tenofovir disoproxil fumarate (TDF) and emtricitabine (FTC). In this study, 64 participants were on ART, where ART regimen was available for 17 individuals (8 – EFV, 4-NVP, 3-lopinavir (LPV) and 1-DTG). Despite the majority of individuals missing ART regimen information, the higher prevalence of NNRTI and NRTI-associated DRM reported in our study supports the exposure of NNRTI and NRTI-containing regimens among these individuals. However, our findings confirmed that individuals with LLV in the settings of HIV-1C can be genotyped, and harbor DRM as reported in other studies. Taken together, these results highlight the concept that genotyping resistance testing may be useful in managing individuals with LLV as resistance data can be used for selecting appropriate ART, especially for those with persisting LLV.

In conclusion, an in-house RNA-based HIV genotyping assay was able to successfully genotype LLV samples (VL 401–999 copies/mL) with the lowest viral load of 423 copies/mL and predicted 17% DRM amongst them. However, the assay can be further optimized to increase the genotyping success rate and for lower VL, and further evaluated for reproducibility and specificity and compared with a commercial genotyping assay.

## Data Sharing Statement

All relevant data are within the paper, including the figures and tables. The sequences obtained in our study were submitted to National Center for Biotechnology Information (NCBI) GenBank and the accession numbers are OP922678 – OP922753.
